# Immunohistochemistry of the circadian clock in mouse and human vascular tissues

**DOI:** 10.20517/2574-1209.2018.46

**Published:** 2018-07-20

**Authors:** Ciprian B. Anea, Ana M. Merloiu, David J. R. Fulton, Vijay Patel, R. Dan Rudic

**Affiliations:** 1 Department of Pharmacology & Toxicology, Medical College of Georgia at Augusta University, Augusta, GA 30912, USA; 2 Department of Surgery, Medical College of Georgia at Augusta University, Augusta, GA 30912, USA

**Keywords:** Circadian blood vessel, vascular endothelium, smooth muscle, Clock, Bmal1, aorta, human, mouse, Per, Cry

## Abstract

**Aim:**

The circadian clock is a molecular network that controls the body physiological rhythms. In blood vessels, the circadian clock components modulate vascular remodeling, blood pressure, and signaling. The goal in this study was to determine the pattern of expression of circadian clock proteins in the endothelium, smooth muscle, and adventitia of the vasculature of human and mouse tissues.

**Methods:**

Immunohistochemistry was performed in frozen sections of mouse aorta, common carotid artery, femoral artery, lung, and heart at 12 AM and 12 PM for Bmal1, Clock, Npas2, Per and other clock components. Studies of expression were also assessed in human saphenous vein both by immunoblotting and immunohistochemistry.

**Results:**

In this study, we identified the expression of Bmal1, Clock, Npas, Per1, Cry1, and accessory clock components by immunohistochemical staining in the endothelium, smooth muscle and adventitia of the mouse vasculature with differing temporal and cellular profiles depending on vasculature and tissue analyzed. The human saphenous vein also exhibited expression of clock genes that exhibited an oscillatory pattern in Bmal1 and Cry by immunoblotting.

**Conclusion:**

These studies show that circadian clock components display differences in expression and localization throughout the cardiovascular system, which may confer nuances of circadian clock signaling in a cell-specific manner.

## INTRODUCTION

This circadian clock is a signaling mechanism that controls 24 h rhythmic oscillations. Heterodimeric interactions of clock transcription factors including Clock, Npas2 with Bmal1 bind E-box response elements of respective genes and drive transcription of downstream negative clock regulators or putative output genes that regulate physiologic function. In the circadian clock loop, the Bmal1-Clock heterodimer serves to cause transcription of Period and Cry genes which are then translated to proteins, form heterodimers themselves, and cycle from the cytoplasm back into to the nucleus to inhibit Bmal1 and Clock. This molecular mechanism is expressed through the body, generating cyclic 24 h physiological rhythms. In blood vessels, the circadian clock is also oscillating^[1–3]^. Since blood vessels are comprised of three distinct cellular layers, the endothelial cell layer, smooth muscle cell layer, and the adventitial layer, circadian clocks may exert cell specific and cell-coordinated functional actions intrinsic within the vasculature. Evidence of vascular cell-specific functions of the circadian clock has first been demonstrated in global knockout models. Global disruption of Bmal1 or Period genes impairs endothelial function and detrimentally influences the adaptation of the vasculature both acutely and chronically^[4–9]^. While the circadian clock is a resilient or robust signaling pathway whereby its disruption is not lethal due to redundancy, intercellular, and intracellular coupling^[10,11]^, overexpression of clock components has been shown to offer protection against deleterious vascular phenotypes^[12,13]^. In cell specific knockout models of the intrinsic vasculature, smooth muscle disruption of Bmal1 results in alterations in rhythmic blood pressure^[14]^, disruption of the circadian clock in the endothelium worsens the thrombogenic response and also affects blood pressure^[15]^, and vascular transplantation of Bmal1-KO mice into WT mice induces arteriosclerotic response^[16]^. In the current study, we sought to examine the cellular expression of the circadian clock in the mouse and human vascular tissue.

## METHODS

### Animals

All animal studies were performed according to protocols approved by the Medical College of Georgia Institutional Committee for Use and Care of Laboratory Animals at Augusta University. Normal wild type C57BL6 mice were used in all experiments.

### Human blood vessels

Segments of intact human saphenous vein were obtained as discarded tissue from other surgical procedures. The procurement of these tissues conforms to the principles outlined in the Declaration of Helsinki and was approved by the human assurances committee of the Augusta University.

### Materials

Tissue sections were probed using the following Antibodies: polyclonal for Bmal1 (Affinty Bioreagents), monoclonal for Clock (Santa Cruz), monoclonal for Npas2 (Abnova), polyclonal for Per1 (Affinty Bioreagents), polyclonal for Cry1 (Novus Biologicals), polyclonal for Rev-erbα (Cell Signaling Technology), polyclonal for Rora (Cell Signaling Technology), monoclonal for Ck1-e (Bectin Dickinson), and polyclonal antibody for Epas (Novus Biologicals), to determine protein expression and localization within the blood vessel.

### Western blotting

Excess tissue from human saphenous veins from patients undergoing coronary arterial bypass surgeries (CABG) were transferred to a dish and kept in EBM2 media (Lonza) in the incubator at 37 °C for further processing at serial time points. Vessels were pooled to permit detection of specific proteins, pulverized on liquid nitrogen, and then immersed into protein lysis buffer.

### Immunohistochemistry

Vascular tissue samples were dissected from regular wild type C57Bl6 mice, and rapidly embedded for frozen cross-sectioning. Sections were cut at 5 μm and mounted onto glass slides. Afterwards, Clock components were immunohistochemically detected. Briefly, the indirect avidin biotin-horseradish peroxidase visualization method was used (ABC Standard and Elite, Vector Red, Vector Laboratories, Burlingame, USA). Samples were incubated with the detection primary antibody at the manufacturer’s recommended concentration.

## RESULTS

**Figure 1:**
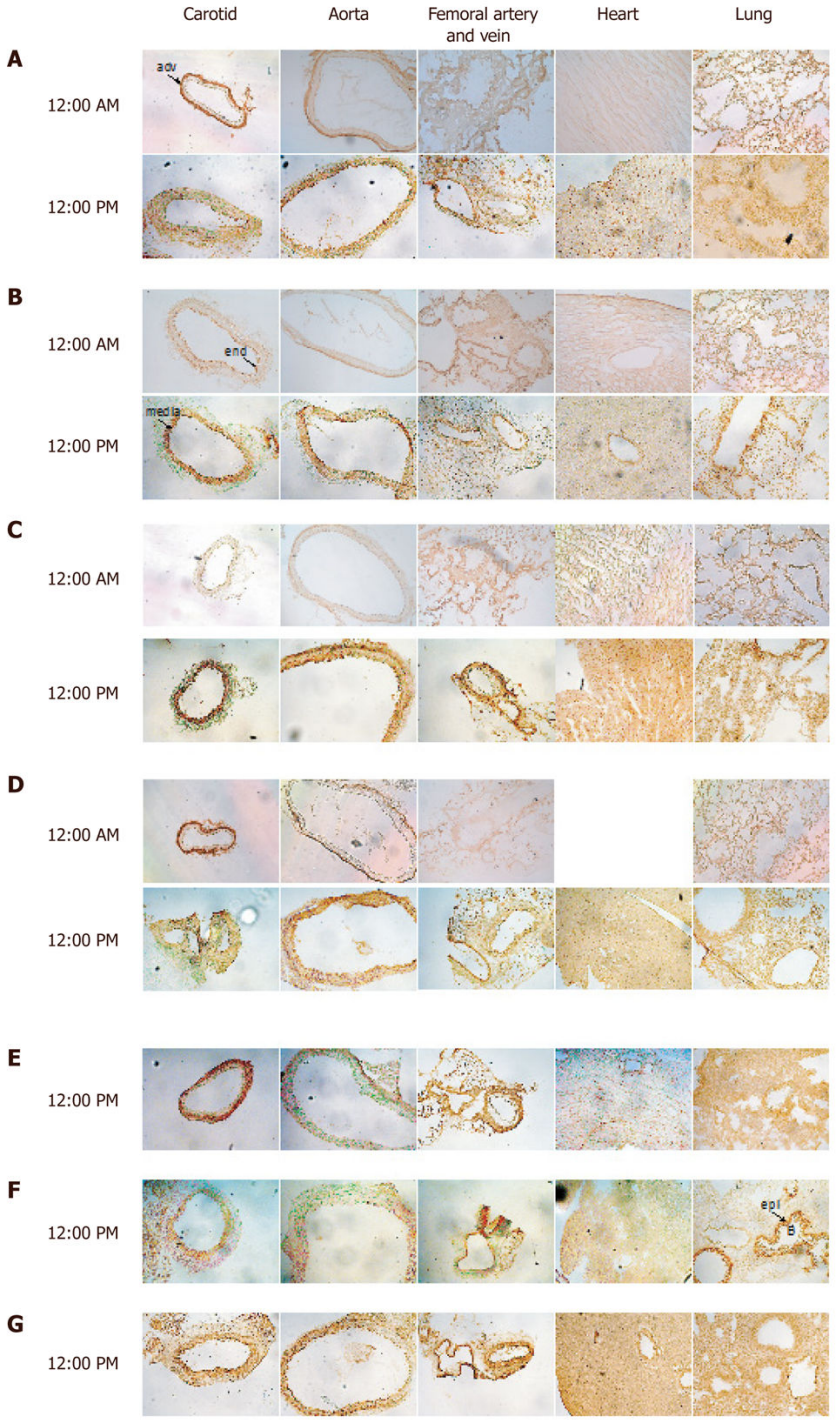
Circadian clock expression in murine cardiovascular tissues. Antibodies to Bmal1 (A), Clock (B), Cry1 (C), Casein kinase (D), Npas2 (E), Rora (F), and Per1 (G) were incubated with frozen carotid artery, aorta, femoral artery/vein, heart, and lung isolated at indicated times of the day to assess localization expression

To determine the cellular and temporal expression of the vascular clock, we harvested tissues from wild-type mice at 12 AM and 12 PM and conducted immunohistochemical analysis of different circadian clock components. At 12 AM, the common carotid artery, aorta, and femoral artery and vein exhibited Bmal1 expression that was largely delineated in the outer adventitial region of the blood vessel [Figure 1A]. Smooth muscle cell expression and endothelial expression were virtually absent. Similarly, at 12 AM, heart and lung exhibited low expression of Bmal1. At 12:00 PM, adventitial Bmal1 expression was reduced, while smooth muscle and endothelial Bmal1 was increased in carotid, aorta, and femoral artery and vein. In heart, there was increased Bmal1 staining that was also evident in lung. Clock staining exhibited enhanced endothelial positivity at 12 AM and was virtually absent adventitial staining in contrast to Bmal1, but did exhibit increased overall tissue expression at 12 PM similar to Bmal1 [Figure 1B]. Cry 1 exhibited little adventitial staining in carotid arteries at either time point, but medial (smooth muscle) staining was increased at 12 PM, as it was also observed in aorta [Figure 1C]. In femoral artery and vein, in contrast to Bmal1 and Clock, Cry exhibited robust expression at 12 PM in the adventitia. In both heart and lung, Cry1 positive cells were robustly increased at 12 PM, with a punctate nuclear stain. In carotid artery and aorta at 12 AM, casein kinase expression was robust, but restricted to the adventitia, and became diffuse in the media at 12 PM [Figure 1D]. Femoral artery, vein, heart and lung followed expression patterns that aligned with the other circadian clock components. Npas2 exhibited a distinctive adventitial staining in all three vessel beds examined, different from the other clock components that occurred at 12 PM, and exhibited punctate nuclear staining in heart tissue and more diffuse staining in lung [Figure 1E]. Ror exhibited medial staining in the blood vessels but most distinctive was in lung tissue, where epithelial cells of bronchioles were highly positive, unique from other clock components [Figure 1F]. Per1 exhibited a strong medial expression in all vascular tissues [Figure 1G, first 3 panels], and also a diffuse expression in heart and lung [Figure 1G, last 2 panels].

**Figure 2:**
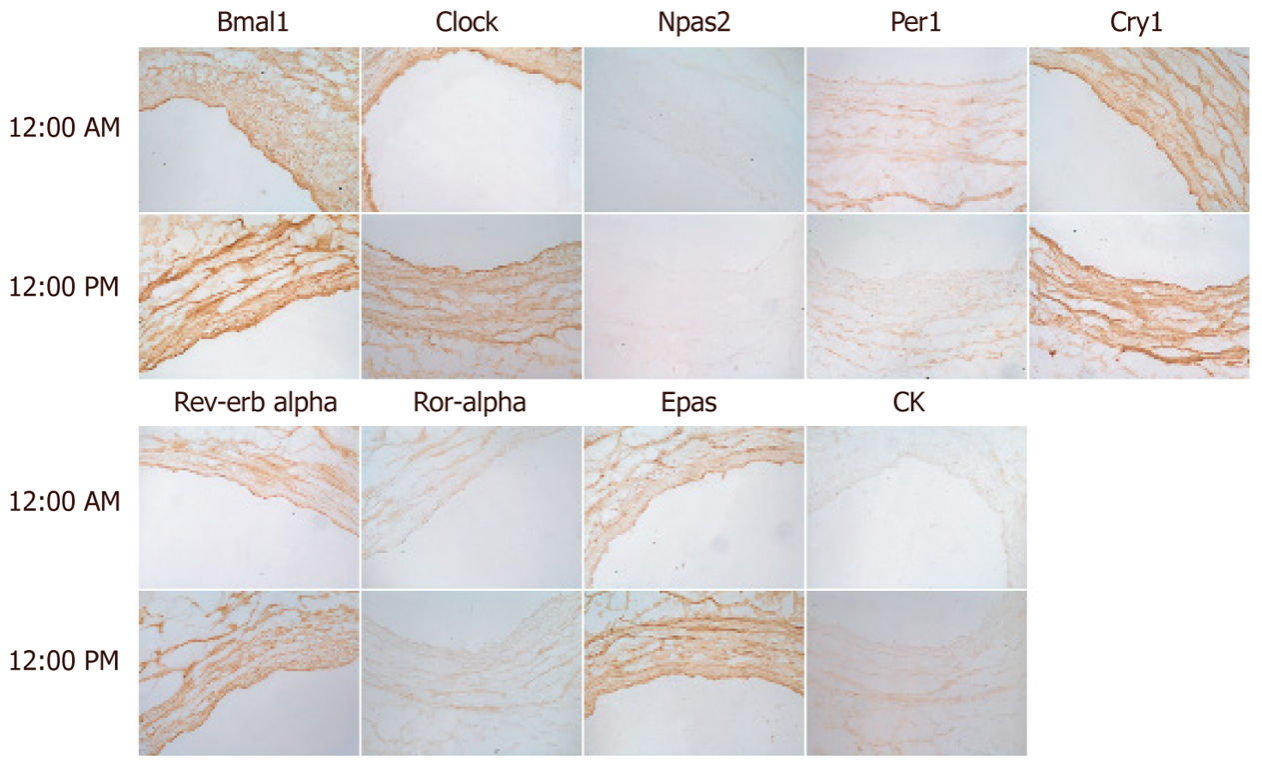
Circadian clock expression in human saphenous vein. Human saphenous veins that remained from coronary artery bypass were immediately procured post-operatively, and incubated in an oxygenated 37 °C incubator. At indicated times, sections of the saphenous vein were frozen in OCT, then sectioned, and incubated with indicated antibodies to the circadian clock

**Figure 3:**
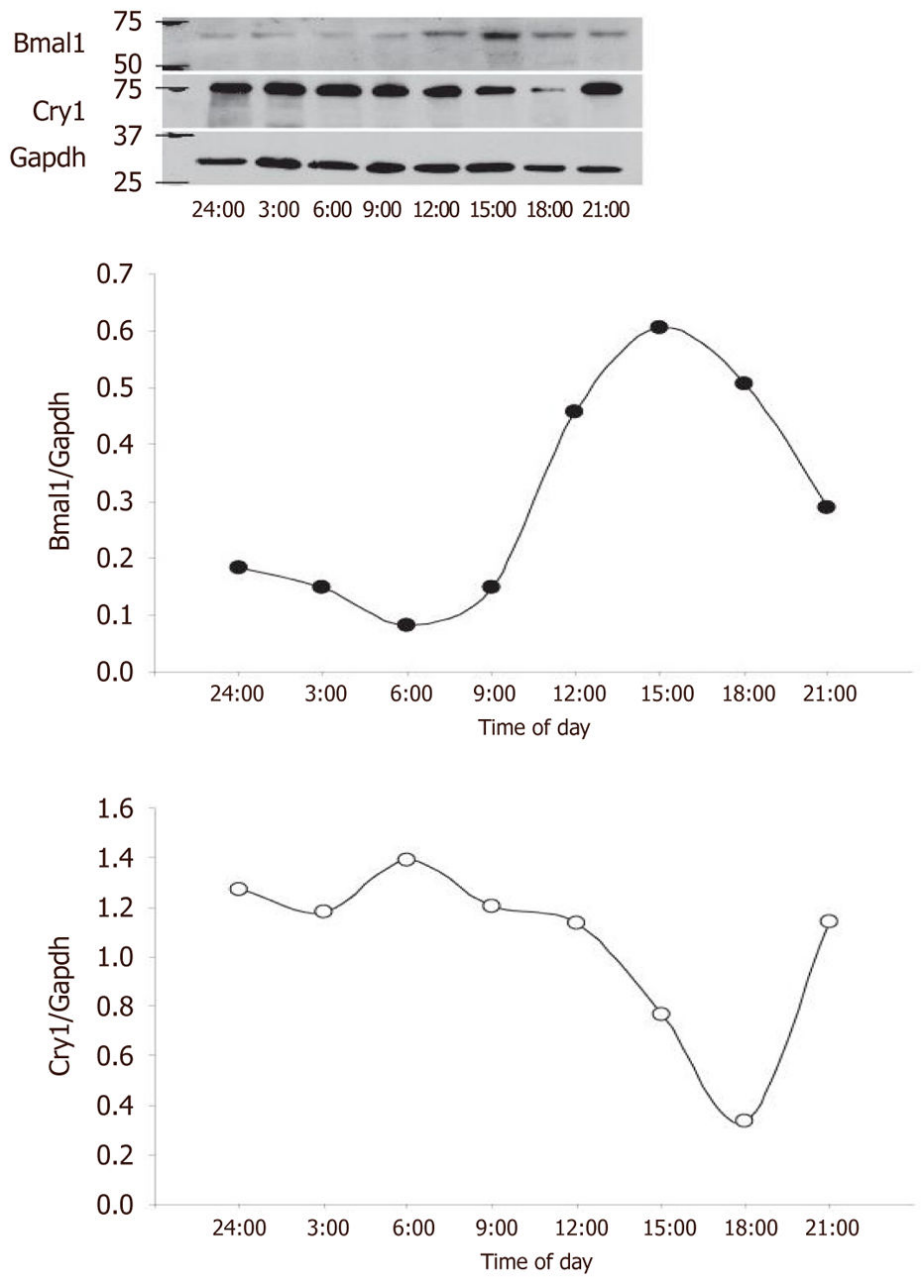
Bmal1 and Cry expression in the human saphenous vein. Human saphenous vein was incubated at 37 °C, and sections were harvested at 3 h intervals from midnight (24:00) to 21:00 (9 PM) for a 21-h time span. At indicated times, saphenous veins were flash frozen and subsequently protein lysates isolated for western blotting (top panel) that was densitometrically quantified (bottom panels)

To complement the study, we next assessed circadian clock expression in the human saphenous vein [Figure 2]. Bmal1 and Clock expression were increased throughout the media at 12 PM relative to 12 AM and exhibited strong endothelium staining. Npas2 staining was relatively absent in the saphenous vein, while Per1 exhibited increased expression at 12 AM relative to 12 PM. Cry1 did not exhibit any temporal difference in expression but was expressed throughout the media. Rev-erbα expression was distributed throughout the media and did not exhibit a temporal expression pattern while Rora and Epas were increased in the media at 12 PM. Casein kinase was not robustly expressed in the saphenous but was increased at 12 PM. We then examined saphenous vein expression of the positive limb component Bmal1 and negative limb component Cry and examined expression by western blot [Figure 3]. Bmal1 trough occurred at 6 PM, while Cry peaked at 6 PM, and Bmal1 peaked at 15:00 (3 PM) while Cry1 was at nadir at 18:00, consistent with the antiphase nature of the positive and negative limb components.

## DISCUSSION

**Figure 4:**
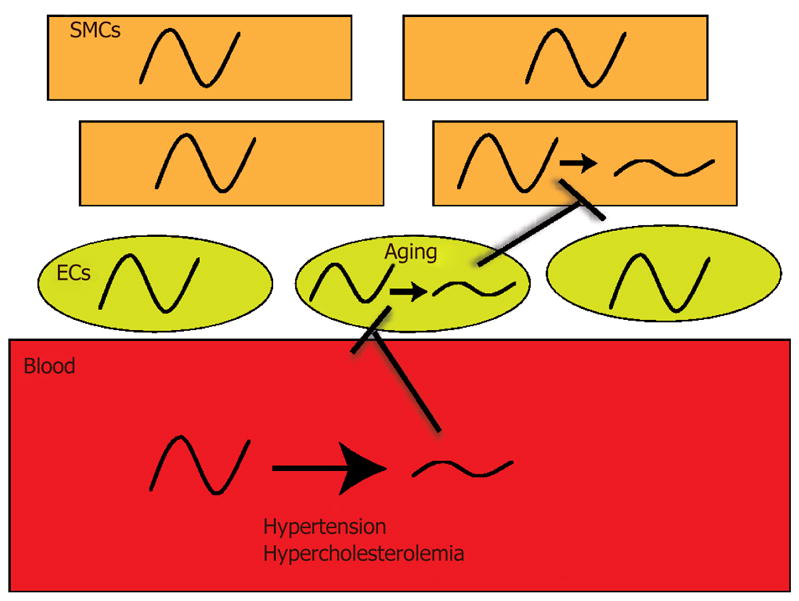
Dysfunctional oscillations in blood, ECs, and SMCs: a potential path to cardiovascular disease. The vasculature has an indirect interaction to the external environment via the bloodstream which is the relay between brain-secreted signals and of curse just eating, for example. Changes in biomechanics and biochemistry od blood could impact underlying endothelial and smooth muscle clocks. Aging and other factors could even impact the ECs and SMCs directly. SMCs: smooth muscle cells; ECs: endothelial cells

The circadian clock is a cell synchronized signaling pathway that serves to control timing. Within the cardiovascular system, heart^[17,18]^, vascular^[2,19,20]^, lung^[21,22]^, and even kidney circadian clocks^[23,24]^ are functional and rhythmic. Because organs are comprised of a heterogeneous population of cells, there are likely cell-specific circadian profiles and expression patterns distinct to the various cell types. Indeed it is known that at the level of the organ, oscillations are different. Central clock oscillation in the SCN is known to oscillate in a different phase than peripheral tissues^[25]^, whereby the SCN is phase advanced to the kidney, which is phase advanced to aorta which is then phase advanced relative to liver^[26]^. The arterial system is composed of three layers; there is an adventitial layer (fibroblasts, pericytes, macrophages)^[27]^, smooth muscle cell layer (media), and an endothelial cell layer (endothelium). The current study examined clock expression in mouse vascular tissue and the human saphenous vein. The approach we elected to use was immunohistochemistry to study clock expression. One key advantage of immunohistological techniques is the ability to differentiate cellular localization. We in particular were interested in endothelial *vs.* smooth muscle, *vs.* the adventitia. To some extent, immunohistochemistry can also provide a rough estimate of nuclear staining or extranuclear staining, but generally distinguishing between subcellular compartments is limited by IHC. In terms of using a fluorescent secondary *vs.* a chromogenic secondary antibody, both are useful, and generally there can be some background fluorescence in tissues with regard to lamina in the vasculature, so we chose the chromogenic susbstrate reaction. In mouse, Bmal1 expression revealed a very strong expression pattern at midnight in the adventitia, in the common carotid artery and aorta and this adventitial staining was decreased at noon. Similarly, casein kinase and Npas2 were also highly expressed in the adventitia. Conversely, media and endothelium staining for Bmal1 was stronger at noon, suggesting that the vascular cell layers are uniquely controlled, which may reflect specific timing of the individual cell-type but may also relate to coordination of paracrine signaling from cell-layer to cell layer. In the femoral artery, adventitial staining was most striking for Npas2 and Cry1. Clock the heterodimeric partner to Bmal1 was not as strongly expressed, but followed a similar temporal profile to Bmal1 in the carotid and aorta in the media. In the heart, all clock components exhibited nominal staining at 12 AM, but expression was robustly increased at 12 PM. In lung, the bronchiole epithelial cells were highly positive for Rora. In the human saphenous vein, Bmal1 exhibited stronger expression at 12 PM *vs.* 12 AM, while Per1 was in antiphase to Bmal1. Similarly, by western blotting, Bmal1 was antiphase to Cry1. Interestingly, we have previously found that endothelial mechanisms such as eNOS and Akt follow or mirror circadian clock expression in particular in regions of altered blood flow^[28]^, while others have demonstrated that eNOS follows the clock in aging^[29]^. In the human veins, the clock was also expressed, and although blood pressure is lower in the venous system than arterial, there is also evidence of a circadian rhythm in blood pressure in the venous system^[30]^. Another potential significance of the circadian clock in the venous system is that it may also relate to disorders such as orthostatic hypotension. Orthostatic hypotension has a prominent circadian component, which may relate to autonomic input dysfunction^[31]^ on both the arterial and venous system, and interestingly has emerged as characteristic in Parkinson’s disease^[32]^. Thus the circadian system may be exerting different functions in the cells within the arterial system and venous system, all which still is largely unknown. Our studies show that circadian clock components display differences in expression and localization throughout the cardiovascular system, which may confer nuances of circadian clock signaling in a cell-specific manner. The bloodstream is a key conduit that relays biomechanical (hypertension) and biochemical information (hypercholesterolemia) from environmental change or disturbance (jet lag, shift work, sleep dysfunction) to the vasculature, while there may also be direct acting clock dampeners such as aging that act on endothelial cells directly to worsen clock function [Figure 4]. These signals may impair function of the clock in ECs to impair other EC’s or to impair SMCs, though it is still not clear if EC clocks communicate with SMC clocks and if there is even EC to EC cell communication. Understanding oscillations of the clock in the cellular milieu of the vasculature will be crucial in delineating how clocks can influence pathology of hypertension and atherosclerosis and ultimately permit the development of improved therapeutic approaches that include timing and clocks into maximizing efficacy and treatment.
